# Virus-Like Particles: Revolutionary Platforms for Developing Vaccines Against Emerging Infectious Diseases

**DOI:** 10.3389/fmicb.2021.790121

**Published:** 2022-01-03

**Authors:** Hasnat Tariq, Sannia Batool, Saaim Asif, Mohammad Ali, Bilal Haider Abbasi

**Affiliations:** ^1^Department of Biotechnology, Quaid-i-Azam University, Islamabad, Pakistan; ^2^Department of Biosciences, COMSATS University, Islamabad, Pakistan; ^3^Center for Biotechnology and Microbiology, University of Swat, Swat, Pakistan

**Keywords:** virus-like particles, emerging infectious diseases, vaccine, vaccine development, expression system, virus, SARS-CoV2

## Abstract

Virus-like particles (VLPs) are nanostructures that possess diverse applications in therapeutics, immunization, and diagnostics. With the recent advancements in biomedical engineering technologies, commercially available VLP-based vaccines are being extensively used to combat infectious diseases, whereas many more are in different stages of development in clinical studies. Because of their desired characteristics in terms of efficacy, safety, and diversity, VLP-based approaches might become more recurrent in the years to come. However, some production and fabrication challenges must be addressed before VLP-based approaches can be widely used in therapeutics. This review offers insight into the recent VLP-based vaccines development, with an emphasis on their characteristics, expression systems, and potential applicability as ideal candidates to combat emerging virulent pathogens. Finally, the potential of VLP-based vaccine as viable and efficient immunizing agents to induce immunity against virulent infectious agents, including, SARS-CoV-2 and protein nanoparticle-based vaccines has been elaborated. Thus, VLP vaccines may serve as an effective alternative to conventional vaccine strategies in combating emerging infectious diseases.

## Introduction

The majority of currently available vaccines are predominantly based on either inactivated (killed) or live attenuated approaches. Although these traditional vaccines have been used effectively against various infectious diseases, some of these have several limitations, which include their lower potential to induce a stronger immune response and poor efficacy (Yan et al., 2020). Recent outbreaks of infectious diseases have manifested the need for the development of robust vaccines to overcome these limitations. The main challenge is to develop new technological approaches that enhance immunity without jeopardizing safety, efficacy, and tolerability. Recent advancements in DNA, mRNA, and recombinant viral-vector based vaccines present effective vaccine development methods for difficult-to-target pathogens and control of infectious disease outbreaks (Francis, 2018; Aida et al., 2021).

**FIGURE 1:**
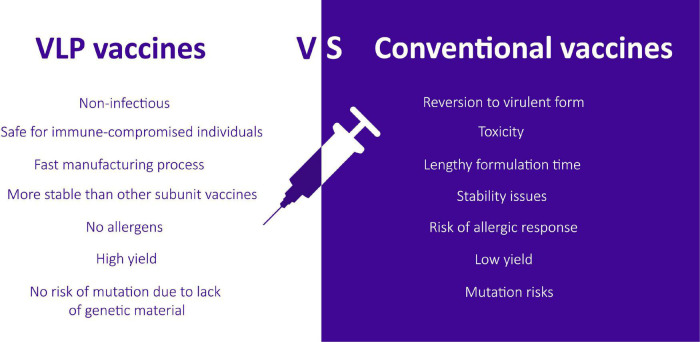
A comparison between VLP-based vaccines and the risks associated with conventional vaccines.

Virus-like particle (VLP) technology presents an alternative platform for developing effective vaccines to combat infectious diseases of serious concern, and it is moving in parallel with mRNA and viral-vector based vaccines (Lampinen et al., 2021). VLPs are also far more immunogenic as compared to other subunit vaccines as they present repetitive antigenic epitopes on their surface in a more authentic confirmation that the immune system can readily detect. Subunit vaccines, on the other hand, have poor immunogenicity due to misfolding of targeted antigen or insufficient presentation to the immunological system (Noad and Roy, 2003). Moreover, they require adjuvants and repeated doses of vaccination to evoke a sufficient immune response (Brisse et al., 2020). Fraenkel-Conrat and Williams (1955) first described the term virus-like particles by reassembling tobacco mosaic virus (TMV) particles from their purified RNA and protein components. The potential of these nanostructures to induce a potent immune response was then further studied later. The VLP platform can overcome various problems that are usually associated with traditional vaccines; specifically, the infectious nature related to live attenuated vaccines, reversion to a virulent form, risk of mutation, reduced immunogenicity of inactivated vaccine, unstable toxicity, low yield, and lengthy formulation time (Mohsen et al., 2018; Figure 1).

**FIGURE 2:**
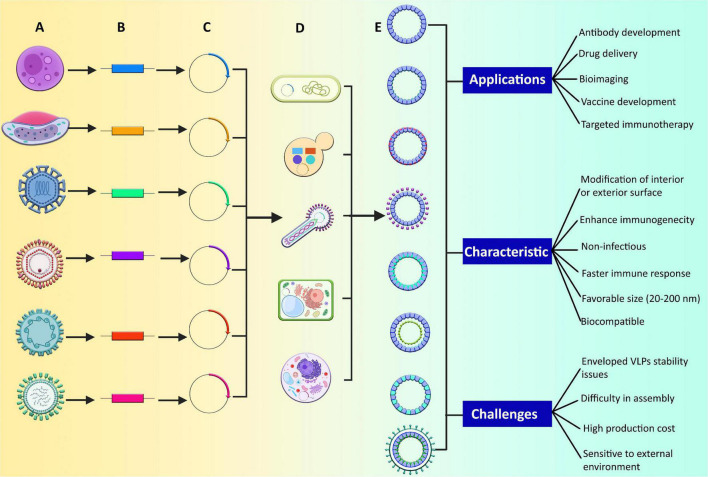
Production of different types of VLPs and their applications, characteristics, and challenges. **(A)** Different human pathogenic viruses and parasites, **(B)** identification of genes that form the structural features of pathogens and can result in the formation of VLPs, **(C)** incorporation of identified genes in expression vectors such as plasmids, **(D)** vectors are allowed to express in various expression systems, **(E)** formation of different VLP types, such as enveloped, non-enveloped, and chimeric VLPs. The non-enveloped VLPs can be of two types: single protein or multiprotein. In multiprotein VLPs, there may be a single layer, multiple layers, and some are mosaic as well. The chimeric VLPs can be modified internally, externally, or can be modified by chemical conjugation.

These bioinspired nanostructures have repetitive and highly dense antigens from different virulent agents that aids in triggering a strong immune response. Moreover, these highly immunogenic molecules have the self-assembling property of viral proteins (Urakami et al., 2017; Pechsrichuang et al., 2021). They are biocompatible and have the potential of structural flexibility during their synthesis (Chung et al., 2020). They can be modified either chemically or genetically and have higher stability, uniformity, and functionality, which are considered an effective tool in various biomedical applications (Qian et al., 2020; Figure 2). They are categorized as enveloped or non-enveloped VLPs based on the presence or absence of a lipid membrane (Jeong and Seong, 2017).

However, some of the key challenges associated with VLPs are lower stability, difficult downstream processing, high production costs, and sensitivity to environmental conditions (Bryant et al., 2007; Vicente et al., 2011). Many diverse VLPs have been synthesized in various expression systems (ESs), such as bacteria, yeasts, mammalian cells, insect cells, and plants (Nooraei et al., 2021). The VLP-based vaccines can potentially be used for the treatment of various infectious diseases, including HIV, influenza, hepatitis B (Spice et al., 2020), hepatitis E, malaria (Qian et al., 2020), Ebola virus (Tripathy et al., 2021), SARS-CoV-2 (Yang et al., 2021), Zika virus, Dengue, and foot and mouth disease, among others (Balke and Zeltins, 2020). Several vaccines based on VLPs have already been licensed and are commercially available in markets including Engerix-B^®^ (GlaxoSmithKline) and Recombivax HB^®^ (Merck & Co) against HBV, Gardasil^®^ (Merck & Co) and Cervarix^®^ (GlaxoSmithKline) against HPV, Hecolin^®^ (Xiamen Innovax Biotech Co.) against HEV (Dai et al., 2018), and Mosquirix™ (GlaxoSmithKline Inc.) against malaria (Mohsen et al., 2017). This review focuses on the basic and advanced technical aspects of the VLP vaccine development. Furthermore, the use of different ESs for VLP production and the development of potential vaccines against various infectious diseases, most notably SARS-CoV-2, have also been discussed. Finally, protein nanoparticles as scaffolds for bearing antigens in the development of vaccines have been elaborated.

## Characteristics of Virus-Like Particles

VLPs, in general, are potential candidates as efficient vaccines because of their distinct characteristics (Figure 2). They are potent immune-stimulatory molecules displaying a highly dense viral surface proteins in an appropriate conformation and a highly repeated way, eliciting strong T and B cell acquired immune responses. Mostly VLPs are derived from viral coat or envelope proteins, although core proteins can also be used (Sarkar et al., 2019). VLPs are naturally biocompatible and not contagious because they lack viral genetic material and hence cannot replicate. They are also thought to be safer (cannot revert to wild type) than conventional live attenuated vaccines (Guo et al., 2019). Moreover, they are highly versatile molecules that varies in their size, with most ranging from 20 to 200 nm. The size range is optimal to drain them freely into lymphatic nodes and for easier uptake by antigen-presenting cells (APCs), particularly dendritic cells (DCs), followed by antigen processing and presentation by major histocompatibility complex (MHC) class II molecules (Syomin and Ilyin, 2019). They are highly organized and can be self-assembled into different geometric symmetry, generally in the form of icosahedral, helical symmetry, rod shape structure, or globular in shape, depending on the virus’s source (Comas-Garcia et al., 2020).

VLPs have been synthesized in a wide range of ESs, including prokaryotic (bacteria) and eukaryotic (insect cells, mammalian cell lines, plant cells, or yeast). The functionality of VLPs can be increased through modifying their exterior or interior surface by displaying the heterologous epitopes of interest using different methods like peptide conjugation, genetic fusion, and chemical crosslinking (Mohsen et al., 2017). The VLP technology offers a significant advantage since it is a faster method of synthesizing vaccines. A new VLP vaccine against a specific strain can be prepared within 12–14 weeks after the strain is sequenced, whereas conventional vaccines usually require 24–32 weeks for the manufacturing processes. These vaccines are free of egg protein, which will give huge relief to individuals who are prone to allergies, as well as stronger protection against diseases than conventional vaccines (López-Macías, 2012).

## Types of Virus-Like Particles Based on Structure

Based on their structural complexity VLPs can be classified into two groups: enveloped and non-enveloped VLPs. Both groups display foreign antigens (Figure 2).

### Non-enveloped Virus-Like Particles

These VLPs are often made up of single or many self-assembled components of the targeted pathogen or viral protein structures. There is no host cell membrane (lipid envelope) in these newly formed VLPs. The expression of the major viral nucleocapsid proteins is primarily responsible for the formation of these VLPs. Non-enveloped VLPs are still being investigated as preferable candidates for developing subunit vaccines against several pathogenic diseases as they are easier to produce and purify (Naskalska and Pyrć, 2015). Furthermore, these VLPs are smaller in size that allows them to easily cross the tissue barrier and drain to lymph nodes (Keikha et al., 2021). The major structural component of the virion is formed by the single, virally encoded protein and is derived from pathogens, such as caliciviruses, papillomaviruses, and parvoviruses (Mohsen et al., 2017), whereas multi-protein non-enveloped VLPs are much more complex containing multiple interacting capsid proteins. They show several striking structural characteristics, such as many complicated concentric layers of different capsid proteins. They are more difficult to make than those that are made up of only one main capsid protein (CP) and derived from infectious bursal disease virus, poliovirus, and retroviruses (Lua et al., 2014).

### Enveloped Virus-Like Particles

In comparison to non-enveloped VLPs, enveloped VLPs (eVLPs) have a far more complicated composition. These nano-structures consist of a cell membrane acquired from the host cell called an envelope, with viral proteins present on the outer surface (Dai et al., 2018). One or more than one glycoprotein spikes are embedded in their lipid bilayers and act as a target antigen for producing neutralizing antibodies. These eVLPs show a higher degree of flexibility as they target antigenic epitopes from the same or heterologous viruses. For example, the eVLPs have been developed that contain Gag protein from SIV and Env protein from HIV (Kushnir et al., 2012). Although it may affect downstream applications because of the presence of the host protein. The eVLPs have been used to develop vaccines against viral diseases, such as the hantaan virus, hepatitis C virus (HCV), influenza A, and retroviruses (Wetzel et al., 2019a). These large eVLPs have a size greater than 100 nm, so there is a chance that they might aggregate at the site of injection and will not reach the lymph nodes that limit their application (Keikha et al., 2021). In comparison, lipid nanoparticles carrying mRNA vaccines have a size range of 100 nm or less and show high efficacy in delivering vaccines to the targeted site (Editorial., 2021; Żak and Zangi, 2021).

### Chimeric Virus-Like Particles

Chimeric virus-like particles (cVLPs) are considered an effective tool for developing vaccines that provide broader, more powerful, and comprehensive protection against emerging infectious diseases (Wetzel et al., 2019a). The complex, multi-protein macrostructures contain epitopes of different viruses (Latham and Galarza, 2001). cVLPs can be created by constructing recombinant DNA molecules that encode both the relevant viral protein and a foreign peptide or protein. These genetically engineered cVLPs display a high number of repetitive sequences on their surface that can be loaded with exogenous antigens from other viruses via chemical conjugation or genetic fusion (Caldeira et al., 2020). Different chimeric vaccines have undergone clinical trials, including the VLP-based vaccine (M2–HBcAg) against hepatitis, an antimalarial vaccine (MalariVax) (Nooraei et al., 2021), anti-influenza A, anti-HIV (Rutgers et al., 1988), and the nicotine-Qb VLP vaccine to reduced nicotine level in the blood of smokers (Maurer et al., 2005). These chimeric particles are advantageous as they substantially increase immune response and antibody titer in response to foreign antigens. Following administration of cVLPs, they induce strong cytolytic T lymphocyte immune responses (Qian et al., 2020). These vaccines are also targeted against non-infectious diseases, such as hypertension, Alzheimer’s, nicotine addiction, allergies, and diabetes (Huang et al., 2017). The upstream and downstream processing yield of cVLPs is usually low, and their *in vivo* stability is also quite uncertain (Buonaguro and Buonaguro, 2014).

## Virus-Like Particles as Immunogens

**FIGURE 3:**
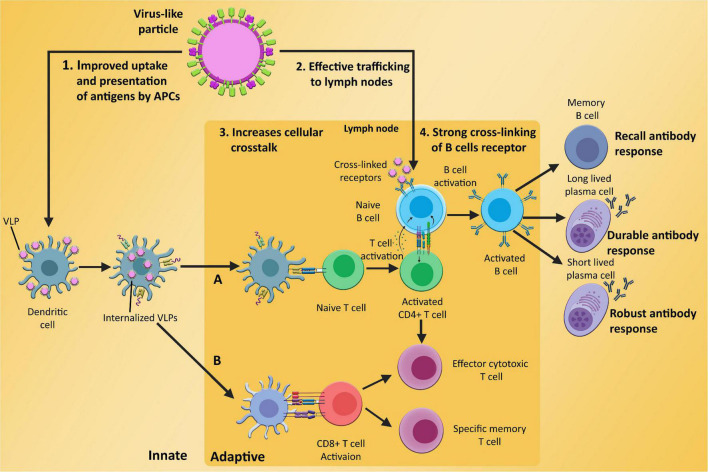
Induction of innate and adaptive immunological responses **(A)** humoral immunity; **(B)** cell-mediated immunity) by VLPs, (1) enhanced absorption and presentation of antigens based on VLP by APCs such as dendritic cells, which inform T cells about potential risks, (2) efficient VLP trafficking to lymph nodes, a crucial site for adaptive immunological responses, (3) improved cellular communication between B cells, T cells, and APCs, and (4) the ability of VLP-based antigen to effectively cross-link and activate B cells receptors, which develop into memory cells and long and short lived plasma cells after antigen exposure.

It has already been demonstrated that VLPs potentially confer high immunogenicity and antigenicity than subunit vaccines. The potency of these particles has the potential to significantly induce cellular and humoral immunity (Mohsen et al., 2017; Figure 3). In response to the VLPs, various maturation markers like CD40, CD80, and CD86, are expressed on the surface of the DCs, which are responsible for the activation of DCs (Quan et al., 2016). In the first step, DCs are activated by binding VLPs to the specific pattern present on the DCs surface called pattern recognition receptors (PRRs) i.e., Toll-like receptors (TLR2) (Sartorius et al., 2021). Following this, internalization of VLPs takes place in the cytosol of DCs and are presented to cytotoxic T cells and helper T cells by MHC class I and class II molecules, respectively (Zepeda-Cervantes et al., 2020; Keikha et al., 2021). VLPs can stimulate not only B cells to mediate antibody response, but they can also stimulate CD4^+^ and CD8^+^ cells proliferation (Roy and Noad, 2008). Some studies show that exogenous antigen can also reach to MHC class 1 pathway through a process called cross penetration (Storni and Bachmann, 2004).

**FIGURE 4:**
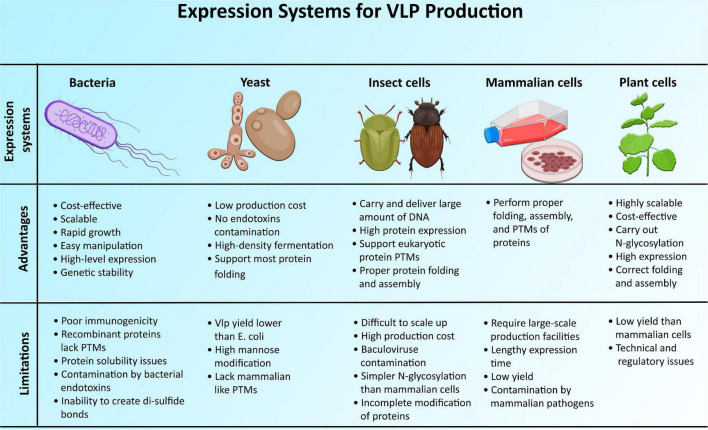
Advantages and limitations of different expression systems for the development of virus-like particles.

Moreover, activation of B cells can induce Th cell expansion and differentiation via toll-like receptor (TLR) signals or homologous interactions, which control the production of cytokines. In order to enhance the efficacy of VLPs, different molecules like Toll-like receptor ligands, biologically active mediators, or other cell receptors can also be attached to VLPs (Keikha et al., 2021).

## Commercially Approved Vaccines Based on Virus-Like Particles

The first recombinant VLPs were synthesized from viral coat protein, two genes from the hepatitis B virus (HBV) (HBsAg and HBcAg), and the tobacco mosaic virus (TMV) (Zeltins, 2013). The first commercial VLP-based vaccine produced by recombinant DNA method was approved by the US Food and Drug Administration (FDA) in 1986s. These are the yeast produced HBsAg vaccines that were named as Recombivax HB^®^ (Lagoutte et al., 2016). Later, in 2006, the second VLP based vaccine Gardasil^®^ against human papillomavirus (HPV) vaccine was licensed by the FDA (Koutsky, 2009). Following this, several VLP vaccines against HPV and HBV have been approved, with some demonstrating efficacy in clinical and preclinical trials. The analysis of studies showed that at least 110 VLPs have been produced from viruses of 35 distinct families (Qian et al., 2020). Several VLP based vaccines targeting different viruses including Norwalk Virus, HIV, Ebola Virus, SARS-CoV-2 Virus, Respiratory Syncytial Virus (RSV), Influenza Virus are still under different clinical trial stages (Nooraei et al., 2021). However, just a few VLP vaccines have made it to the market, showing their commercial feasibility, and majority of them are effective against non-enveloped viruses (Guo et al., 2019).

## Challenges Specific to Virus-Like Particle-Based Vaccine Platforms

At Present, VLPs are as effective as conventional vaccines, with the additional benefit of being safer. Nevertheless, several obstacles in the successful development of VLP-based vaccines need to be addressed. The main challenge is to identify issues related to downstream processing in the production of clinically viable VLPs (Charlton Hume et al., 2019) for their timely management and economic feasibility.

### Stability of Enveloped Virus-Like Particles

VLPs are generally considered more stable than subunit vaccines (Dai et al., 2018). However, when the environmental conditions change, especially during downstream processing, VLPs can become very unstable as they lack the genetic material of the virus. Despite the fact that multiple VLP vaccines are already available in the marketplace, some of the candidates’ vaccines have stability issues (Mohsen et al., 2018). Generally, eVLPs are often more susceptible to external environmental conditions as compared to non-enveloped VLPs (Dai et al., 2018). Variations in conditions such as a change in temperature, shear stress, dissolved oxygen, fluid dynamics, agitation rate, and chemical treatment may all have an impact on the particle’s integrity and stability (Roldao et al., 2011). Moreover, this structural breakdown significantly reduces the immunogenicity of eVLPs. It also interferes with cell growth and the production of metabolic proteins, which has an impact the VLP production. It has been one of the key obstacles to using them as an alternative for a live virus in vaccine manufacture. However, several modifications have been made to increase their thermostability. One typical procedure is the insertion of stabilizing mutations (Dai et al., 2018). A study conducted by Marsian et al. (2017) showed that when stabilizing mutation was induced within the coat proteins of poliovirus type 3 VLP, it become more stable than wild type VLPs. It modified capsid precursor and the viral protease without disturbing its antigenic epitopes and structural confirmation. In another study stability of chimeric (HBcAg-VLP) was increased by the addition of C-terminal linker-hexahistidine-peptide (Schumacher et al., 2018).

### High Production Cost

Some VLP-based vaccines are much more complex and, as a result, have a higher cost of production. The numerous impurities associated with eVLPs pose a daunting challenge. During downstream processing (DSP) various impurities like host cell debris (HCD), host cell protein (HCP), and host cell DNA (HCD) are co-purified as in the case of enveloped baculovirus particles. These contaminations can cause undesirable side effects in vaccines if not removed properly during DSP (van Oers, 2011). Large scale production and purification of VLPs require different processes like density gradients or even chromatography to make the final formulated product. These complex processes are very costly and time-consuming (Wetzel et al., 2019a). This also leads to difficulty in industrial scale production and requires several quality control efforts as various downstream processing steps may worsen the VLPs quality (Diamos et al., 2019). A more robust and better analytical methods are required to ensure the quality and quantity of the product that can facilitate their clinical and pharmaceutical utilization (Moleirinho et al., 2020). Different strategies such as clone screening, high-throughput screening, bioreactor engineering, material/matrix screening, filtration, flow-through or size-exclusion chromatography, and polishing have been implemented during upstream and downstream processing for scalable and cost-effective industrial manufacturing of VLPs (Vicente et al., 2011; Lagoutte et al., 2016).

### Difficulty in Assembly

The genetic fusion of the sequences of epitope into VLPs can sometimes be challenging, as VLPs may lose their self-assembly property or cause particle misfolding (Guo et al., 2019). Genetically fusion of antigen to capsid protein of virus often hinders either antigen assembly or VLP, making the technique laborious. Therefore, time-intensive planning and optimization are required for individually testing every single antigen (Lampinen et al., 2021).

## Expression Host Systems for Virus-Like Particle Production

Different expression systems (ESs) were utilized to make VLP vaccines, including plant, mammalian, insect, yeast, and bacteria (Figure 4).

### Bacteria

Most of the bacterial systems are focused on well-studied industrial strains and expression vectors of *Escherichia coli* (Zeltins, 2013). Bacterial cell cultures were investigated as a platform for VLP development, with benefits in terms of cost and scalability (Kim et al., 2014). The other beneficial features of using bacterial systems for VLP production include (a) easy manipulation, (b) high-level expression, (c) fast growth rate, (d) genetic stability, and (e) simplicity of expression (Masavuli et al., 2017).

Huo et al. (2018) used pCold expression vector to express and purify norovirus (NoV) VLPs *in E. coli* strain (BL21) and demonstrated the similar binding pattern for VLPs assembled in *E. coli* as for Sf9 cells assembled VLPs. Similarly, in a recent study, the full-length CP of type 2 porcine circovirus and VP2 protein of porcine parvovirus were expressed in *E. coli*, which were self-assembled into VLPs. The study suggested that the expression of the CP and VP2 in *E. coli* is possible for the mass development of VLP vaccines (Liu et al., 2020). Yazdani et al. (2019) investigated the possibility of utilizing VLPs of grapevine fanleaf virus (GFLV) as a potential vector for presenting the L2 epitope of HPV. The antigenic determinant sequence was incorporated genetically into the GFLV capsid protein’s “αB-αB” domain C, which was then overexpressed in *E. coli* and *Pichia pastoris*. In *E. coli*, the highest expression yield was observed. For Hepatitis E virus (HEV), the ORF2 protein region 368–606 aa was purified *in vitro* from the insoluble *E. coli* fraction that is assembled into VLPs. This HEV VLP promises 100% effectiveness in clinical trials against symptomatic HEV and is approved as a vaccine for commercial use in China (Gupta et al., 2020). In another study, infectious hypodermal and hematopoietic necrosis virus (IHHNV) VLPs from CP of recombinant IHHNV were reconstructed in *E. coli* and showed excellent physical stability (Kiatmetha et al., 2018).

Bacterial systems are not always the optimal VLP development strategy because of several factors, including (a) poor immunogenicity, (b) inability to develop recombinant proteins with mammalian−like post-translational modifications (PTMs), (c) issues of protein solubility, (d) inability to create the correct disulfide bonds, and (e) presence of bacterial endotoxins/or lipopolysaccharides in preparation of recombinant proteins (Shirbaghaee and Bolhassani, 2016; Masavuli et al., 2017).

Brito and Singh (2011) have highlighted the acceptable level of endotoxins for various types of vaccines. There are various methods by which endotoxins are removed during the purification step, such as immobilized sepharose, surfactants, activated carbon, ultrafiltration, and anion exchange chromatography. However, the use of these techniques often leads to a considerable reduction in yield, an increase in cost, or a lack in the biological activity of the target protein. Therefore, ClearColi™, a genetically engineered *E. coli* strain with a genetically modified LPS which does not elicit an endotoxic reaction in humans, has been developed (Mamat et al., 2013).

### Yeast

Eukaryotic ESs are a compelling alternative to prokaryotic ones, particularly when it comes to addressing the issue of bacterial endotoxins in vaccine production (Shirbaghaee and Bolhassani, 2016). VLPs can be produced more cost-effectively by recombinant expression of proteins in yeast cells. Manipulation of genes in yeasts is comparatively straightforward, and the transformed cells can grow to extremely high densities until the expression of recombinant proteins is induced. This allows commercial-scale fermenters to produce VLPs in large volumes (Stephen et al., 2018). Some mammalian viruses’ structural genes expressed in yeast can form the VLPs (Shirbaghaee and Bolhassani, 2016).

The FDA has approved some yeast-derived VLP vaccines, including Gardasil^®^ and Gardasil9^®^ against HPV and Mosquirix™ against P. falciparum (Nooraei et al., 2021). Some studies have investigated VLP production using yeast as an ES. Wetzel et al. (2018) established a robust and cost-effective yeast model for the production of cVLPs. The duck HBV membrane integral small surface protein (dS) was selected as the scaffold for VLP, and the safe and industrially applied Hansenula polymorpha yeast as the heterologous expression host. 8 distinct antigens of high molecular weight were derived from four viruses that infect animals and are genetically linked to a protein dS, and then the recombinant isolates were identified and purified. The fusion proteins were highly expressed in all cases, and it was possible to generate chimeric VLP comprising both proteins after co-production with protein dS. The production system based on yeast allows for a low-cost product that is not restricted to small-scale basic research. In another study, Alireza et al. (2018) produced chimeric protein L1/L2 VLPs in the *P. Pastoris* system by first inserting a cross-neutralizing epitope from the gene HPV-16 L2 into the gene L1 HPV-16. Following that, the chimeric L1/L2 HPV-16 had been introduced into the plasmid (pPICZA) and expressed in *P. pastoris*. The ELISA results for L1-HPV-16 Ab as well as L2-HPV-16 Ab detection indicated a positive reaction with sensitivity comparable to the commercial testing kit. Similarly, in the *P. pastoris* ES, Gupta et al. (2020) produced a recombinant VLP against HEV that included 112–608 aa region of the protein ORF2. The results showed that for the development of 112–608 aa VLP, the *P. pastoris* ES seems to be a superior and safer alternative to the baculovirus (Bv) ES.

During viral infection, enteroviruses like poliovirus, generate empty capsids that are antigenically indistinguishable from that of mature virions. The recombinant synthesis of such capsids with the help of heterologous systems like yeast has enormous potential as candidates for the VLP vaccine. Sherry et al. (2020) showed VLP production in *P. pastoris* through co-expression of the viral protease 3CD and the structural precursor protein P1.

Construction of yeast ESs is much more challenging than bacterial ESs, especially the Pichia and Hansenula strains. Furthermore, the VLP yield is lower than that of *E. coli*. Another disadvantage of the yeast ES is its lack of resemblance to mammalian ESs in protein PTMs, particularly glycosylation (Shirbaghaee and Bolhassani, 2016). Their glycoforms are mostly of the high type of mannose, which is undesirable for most pharmaceutical glycoproteins (GPs) (Kim et al., 2014).

### Insect Cells

Baculovirus-based protein expression in insect cell lines has appeared as an effective tool for developing complicated protein-based biologics for a variety of purposes, extending from multiprotein complexes to the development of proteins for therapeutic use, such as VLPs (Sari-Ak et al., 2019). Bv is unusual among widely used viral vectors in its ability to tolerate heterologous DNA in large amounts and deliver it faithfully to the desired host cell (Gupta et al., 2019). Gopal and Schneemann (2018) described detailed procedures for producing recombinant baculoviruses (rBVs), screening for VLP expression in insect cell lines, and purifying VLPs.

The Bv-insect cell system is a two-step procedure in which insect cells are originally grown to the required cell concentration before being infected with rBVs for protein expression (Roldão et al., 2010). Recombinant protein and VLP production using Bv expression vector systems (EVSs) is fast, versatile, and provide substantial yields (Strobl et al., 2020). This system can produce exceptionally high protein production levels, combined with complex eukaryotic protein PTMs, which may be crucial for the proper self-assembly and release of some VLPs (Chaves et al., 2018).

MultiBac is an innovative Bv EVS that comprises of a viral genome that has been engineered to suit specific purposes. Recently, a team of scientists described the development of a MultiBac-based VLP-factory, dependent on the CP (M1) of influenza virus and its utilization in generating an arsenal of influenza-derived VLPs with functional modifications in influenza virus hemagglutinin (HA), which are expected to regulate the VLP-derived immunological response (Sari-Ak et al., 2019).

The insect cell-Bv EVSs have proven to be as effective as conventional egg- and cell-based approaches in influenza virus vaccine production with additional features like high production yields, and short production times. Moreover, rBVs construction has become faster, easier, and more flexible, allowing for the fusion of genes from different types and/or subtypes of influenza viruses inside the same expression vector. Sequeira et al. (2018) successfully created a robust High Five cell-based insect platform that combines stable expression with Bv-mediated expression to generate multivalent influenza VLPs. The capability of this modular approach has been proven by infecting the High-Five cell line with 2 distinct HA proteins of subtype H3 (called HA2 and HA1) with a Bv expressing M1 and 3 additional HA proteins of subtype H3 (called HA5, HA4, and HA3), to generate pentavalent VLPs (H3).

The Zaire Ebola virus serotype (ZEBOV) is the most virulent and has the highest mortality rates among other serotypes. ZEBOV-VLPs development have been achieved in insect and mammalian cell lines via co-expression of 3 viral structural proteins, the nucleocapsid protein (NP), the matrix structural protein (VP40), and the glycoprotein (GP). A technique for generating ZEBOV-VLPs in insect cell line was reported by Pastor et al. (2019), which basically consists of employing a high multiplicity of infection (MOI) of bac-GP and bac-VP40, and limiting the NP expression, either via preventing infection or by lowering the bac-NP MOI, was the most suitable for developing VLP.

The main limitations of the insect cell ES are (a) protein contamination by enveloped baculoviruses, (b) difficult to scale-up, and (c) simpler N-glycosylation than mammalian cells (Shirbaghaee and Bolhassani, 2016; Masavuli et al., 2017).

### Mammalian Cells

For more than two decades, various mammalian cell lines such as murine myeloma (Sp2/0, NS0), Chinese hamster ovary (CHO), murine C127, baby hamster kidney (BHK21), HT-1080, and HEK293 were established as a possible source of commercialized protein therapeutics for medical purposes, due to their capability to properly fold, assemble, and post-translational modification to proteins (Dumont et al., 2016; Shirbaghaee and Bolhassani, 2016). Systems based on mammalian cell culture offer many benefits, including consistency and flexibility during the development process. It also helps glycosylated proteins to be recovered with compositions of lipid membrane similar to the virus’s host. It has been reported that the stable transfection of viral genes into mammalian cell lines, including 293 or Vero cells, results in VLP production (Buffin et al., 2019).

Several studies reported the efficient production of VLPs from mammalian cell cultures. Hsin et al. (2018) developed a MERS VLP system using Huh7 cells as an expression system for understanding virus infection and morphogenesis. In another study, influenza VLPs encoding neuraminidase (NA), hemagglutinin (HA), and matrix M1 proteins had been expressed in Vero, 293 T, or CHO-K1 cell lines, using transient transfection. Preclinical studies in BALB/c mice revealed that influenza VLPs, when given intramuscularly, were significantly immunogenic at low dosages, inducing functional Abs against NA and HA (Buffin et al., 2019). Wu et al. (2010) designed a scalable method for the effective production of various subtypes of influenza VLPs expressed in mammalian cell line. The study demonstrated that these mammalian influenza VLPs were very similar to the original viruses in particle size, structure, host factor composition, and viral antigen glycosylation. Similarly, in another work, an inducible cell line of human embryonic kidney HEK-293 expressing NA and HA was developed and utilized to generate VLPs following transient transfection with a plasmid encoding HIV-1 Gag (Venereo-Sanchez et al., 2017). A protocol developed for the synthesis of HIV-1 Gag VLP in mammalian cell suspension cultures via transient gene expression showed that the large proportion of Gag-GFPs present in the supernatants of cell culture was fully assembled into VLPs of the predicted morphology and size consistent with immature particles of HIV-1 (Cervera et al., 2013).

Expression systems based on mammalian cells require large-scale production facilities, like fermentation bioreactors, which are prohibitively expensive to construct. As a result, the high cost of production is a challenging part of the cell-based mammalian ES (Kim et al., 2014). The other limitations include lengthy-expression time, low yield, and vulnerability to infections with mammalian pathogens (Masavuli et al., 2017).

### Plants

“Molecular farming” is a term used to describe the utilization of plants or plant cells to produce recombinant proteins or other biologic drugs for use as cosmeceuticals, biopharmaceuticals, therapeutics, vaccines, and other reagents (Rybicki, 2020). Plants provide an enticing alternative system for the manufacture of VLP vaccines due to their potential to generate significant amounts of recombinant proteins at a minimal cost, their eukaryotic production machinery for the PTM and correct protein folding and assembly, and their low risk to introduce adventitious human pathogens. Plants do not demand the installation of costly fermentation facilities for the production of biomass, nor do they necessitate the establishment of duplicate facilities for scale-up production. Unlike microbial fermentation, plants have the ability to carry out N-glycosylation as a glycoprotein PTM (Chen and Lai, 2013). Engineered VLP-forming human or animal virus capsid proteins expressed in plant cells include human norovirus CP, HPV L1 protein, hepatitis B core and surface antigens, HIV Gag polyprotein, and HA protein of influenza virus (Rybicki, 2020).

Several studies have extensively discussed the concepts for constructing VLPs in various plant ESs, efficient growth of VLPs in plant hosts, and the self-assembly of multiple structural proteins of viruses in plants. Santi et al. (2008) produced properly assembled recombinant Norwalk virus VLPs in leaves of Nicotiana benthamiana utilizing a TMV-derived transient ES. Young et al. (2015) generated trackable hemagglutinin based VLPs that allowed them to examine particle assembly in plants and the interaction of VLPs inside the mammalian immunological system. van Zyl et al. (2016) investigated the production of bluetongue virus VLPs in N. benthamiana through Agrobacterium-mediated transient expression, which is an inexpensive system. Similarly, Veerapen et al. (2018) transiently expressed VLPs of the foot-and-mouth disease virus in N. benthamiana. Diamos and Mason (2018) produced norovirus VLPs in a plant-based system using modified geminiviral vectors. Recently, Rosales-Mendoza et al. (2020) presented a perspective in developing VLP vaccines based on plants against SARS-CoV-2, which is responsible for the COVID-19 pandemic.

Plants for the production of the VLP platform are not entirely acceptable due to comparatively lower production levels of VLP than mammalian ESs and plant-specific N-glycosylation of glycoproteins (Kim et al., 2014). Nonetheless, the recent creation of novel plant ESs, as well as advancements in plant glycoengineering, have both resolved these challenges (Chen and Lai, 2013). Recent advancements in plant glycoengineering permit human-like modification of glycol and optimization of desirable glycan structures to improve the functionality and safety of recombinant pharmaceutical glycoproteins (Kim et al., 2014).

## Development of Virus-Like Particle Vaccines Against Emerging Infectious Diseases

Several VLP vaccines have been produced and are being used against different viral and parasitic infections in recent years (Table 1). The development of VLP vaccines against zika virus, chikungunya virus, influenza virus, and human papillomavirus have been discussed below.

**TABLE 1:** VLP vaccines against different viruses and parasites.

VLP vaccine	Antigens displayed by VLP vaccine	Expression system	Targeting pathogen	Mechanism of action	References
M-HBsAgS-N4, M-HBsAgS-N9 VLPs	NANP repeats from circumsporozoite protein (CSP) and small HBV envelope protein (HBsAgS)	HEK 293F cells	*Plasmodium falciparum*	Induced anti-NANP Abs with the potential to initiate the complement system, which led to the inactivation of invading parasitic sporozoites.	Kingston et al., 2019
STh and STh-A14T VLPs	Human heat-stable toxins (STh) and STh-A14T toxoid	*E. coli*	Enterotoxigenic *Escherichia coli* (ETEC)	Both VLPs showed immunogenicity in mice and neutralized the native STh’s toxic activities completely.	Govasli et al., 2019
CV-B4 VLPs	VP1	Insect cells	Coxsackievirus B4 (CV-B4)	Showed antigenic reactivity with specific antibodies.	Hassine et al., 2020
RVFV VLPs	Gn, Gc, and N proteins	Sf9 insect cells	Rift Valley fever virus (RVFV)	Produced RVFV neutralizing antibodies in mice and stimulated spleen cells in the mouse to produce high cytokines levels (IL-4 and IFN-γ).	Li et al., 2020
Genogroup II, genotype 17 (GII.17) VLPs	Major capsid protein (VP1)	sf9 insect cells	Noroviruses (NoVs)	Mice immunized with purified and sterile VLPs developed specific GII.17 sera and effectively blocked GII.17 VLPs bound to antigen of the saliva histo-blood group.	Chen et al., 2020
JEV genotype III (GIII) VLPs	Envelope (E) protein and Precursor membrane protein (prM)	Mosquito cell lines	Japanese encephalitis virus (JEV)	A specific immune response has been developed against a stable IgG2a/IgG1 ratio. This response essentially nullified both Japanese encephalitis virus GIII and GI and triggered a hybrid response of Th1/Th2 in a mice model.	Chang et al., 2020
SAG1-VLPs	Surface antigen 1 (SAG1)	Sf9 insect cells	*Toxoplasma gondii*	After immunization, IgG, IgG1, IgG2a, and IgA were significantly enhanced, and *T. gondii* endurance rates were severely constrained by the immunized sera.	Choi and Park, 2020
VLP-gG and VLP-gB	ILTV glycoproteins B (gB) or G (gG)	LMH cells	Infectious laryngotracheitis virus (ILTV)	VLPs displayed no noticeable adverse effects *in vivo* and appeared to induce an antibody-based immune response.	Schädler et al., 2019
Chimeric VLP (Pfs230 and Pfs25), genetically fused to dS of the duck HBV	Pfs25 and Pfs230	Auxotrophic *Hansenula polymorpha* strain ALU3	*Plasmodium falciparum*	Exhibited reactivity with transmission-blocking antibodies and established the malaria antigens exhibition on the native VLP surface.	Wetzel et al., 2019b
Triple chimeric AHSV-6 VLPs	VP2, VP3, VP5, and VP7	*Nicotiana benthamiana* dXT/FT plants	African horse sickness virus (AHSV)	Able to stimulate a poor neutralizing humoral immune response against homologous AHSV virus in target animals.	Rutkowska et al., 2019
Codon-optimized AMA-1 VLP	Apical membrane antigen 1 (AMA-1)	Sf9 insect cells	*Plasmodium berghei*	Vaccination with codon-optimized AMA-1 VLPs, mediated elevated levels of B cells, CD8^+^ T cells, germinal center cells, and CD4^+^ T cell responses relative to non-codon optimized VLPs.	Lee et al., 2019
HBc_ΔR82_, HBc_ΔH301_, HBc_ΔH82_, and HBc_Δ R301_ VLPs	CD4^+^ cell epitope (AS15), B cell epitope (SAG1_301–320_ or SAG1_82–102_), and a CD8^+^ cell epitope (ROP7 or HF10)	*Escherichia coli*	*Toxoplasma gondii*	High titers of IgG Ab and production of interferon (IFN)-p, resulted in reduced brain parasite load.	Guo et al., 2019
PPRV VLPs	Hemagglutinin (H), PPRV matrix (M), nucleocapsid (N), and fusion (F) proteins	Baculovirus-insect cell	Peste des petits ruminants virus (PPRV)	Induced antibodies production specific for F and H proteins and provoked a cellular immunological response in goats.	Yan et al., 2019
EV71-VLPs	VP0, VP1, and VP3	*Pichia pastoris*	Enterovirus 71 (EV71)	Both maternally transferred Ab and passive transfer protection mouse models stimulated a robust neutralizing Ab response and offered effective protection against lethal challenge.	Yang et al., 2019
CJaYZ vaccine	CprME-IRES-NS2B-3, (C-E3-E2-6K-E1)	293?T stable cell lines	ZIKV, CHIKV, JEV, and yellow fever virus (YFV)	The tetravalent VLPs supplied highly neutralizing Ab titers against the viral strains tested.	Garg et al., 2020
Chimeric BTV-4 and BTV-3 VLPs	VP3, VP7, VP2, and VP5	*N. benthamiana*	Bluetongue virus (BTV)	Induced long-lasting serotype-specific neutralizing Abs in sheep like the monovalent live attenuated vaccine controls.	Mokoena et al., 2019
AP205 capsid-based VLPs	The VAR2CSA PM antigen and HPV RG1 epitope	*E. coli*	Human Papillomavirus and placental malaria	Reduced *in vivo* HPV infection and induced IgG antibodies against VAR2CSA.	Janitzek et al., 2019
CVB1-VLPs	CVB1 capsid proteins (VP0, VP1, and VP3)	Baculovirus-insect cell	Type B Coxsackieviruses (CVBs)	CVB1-VLP vaccines were extremely immunogenic, and their immunogenicity and stability improved with formalin treatment.	Hankaniemi et al., 2019
HCV VLPs	E1 and E2 glycoproteins	Huh7 cells	Hepatitis C virus (HCV)	Produced robust HCV multi-genotypic neutralizing Ab (NAb), as well as cell mediated immunity responses in pigs.	Earnest-Silveira et al., 2016; Christiansen et al., 2019
Hepatitis B core (HBc) VLPs and Recombinant immune complexes (RIC)	Minor CP (L2 or L2 fused with an immunoglobulin)	*N. benthamiana*	Human Papillomavirus (HPV)	Both candidates for the vaccine showed potent immunogenicity in a mice model but were particularly so when delivered together, producing very high and consistent HPV L2-directed antibody titers, which associated with the neutralization of viruses.	Diamos et al., 2019

### Zika Virus

Zika virus (ZIKV) is a small-enveloped mosquito-borne neurotropic positive-strand RNA virus of the family Flaviviridae (Lin et al., 2018; Shanmugam et al., 2019). ZIKV infections were associated with acute prenatal abnormalities like microcephaly in neonates born to infected mothers, and also Guillain-Barré syndrome (GBS) in adult people (Alvim et al., 2019). Currently, no cure or vaccine is commercially available for effective therapy or treatment, so vaccine development against ZIKV is of great importance (Boigard et al., 2017).

ZIKV has an RNA genome with only one open reading frame, and a solitary polyprotein is formed. This protein-strand is cut into 7 non-structural proteins (NS5, NS4B, NS4A, NS3, NS2B, NS2A, and NS1) and 3 structural proteins (C, prM, and E) by cellular and viral proteases. E-protein is engaged in the binding of viral particles as well as its fusion (Boigard et al., 2017). E-protein has three domains structure, like enveloped-domain-I also known as ED-I, then ED-II, and ED-III. Humoral response mainly targets the fusion-loop belonging to ED-II. Antibodies against fusion-loop epitopes enhance the uptake of DENV by Fcγ-receptors (Shanmugam et al., 2019). So, this protein may serve as a possible target for vaccine development (Shanmugam et al., 2019).

Many VLPs for ZIKV have been developed (Boigard et al., 2017). Garg et al. (2019) compared different VLPs-vaccine for ZIKV in mice and showed that CprME-VLPs (Capsid-preMembrane-Envelope) gave better results than prME-VLPs (preMembrane-Envelope). Cell lines could not be generated using CprME- because co-expression of protease NS2B-3 is needed. In order to get rid of this barrier, a bicistronic vector was generated that uses IRES-sequence to produce both NS2B-3 and CprME-VLPs. Alvim et al. (2019) demonstrated the continuous expression of ZIKV-VLPs by HEK293-cells. In short, the cell lines constitutively producing Zika-VLPs are ideal for developing a vaccine.

### Chikungunya Virus

It’s a severe and periodic infectious disease caused by CHIKV (chikungunya-virus) transmitted by a carrier mosquito. Symptoms are high fever, skin rash, etc. No vaccine is available at present, but many VLP-vaccine candidates are under development in different stages (Zhang et al., 2019). Protection from multiple strains of this virus can be conferred by self-assembled VLPs produced as a result of selective expression of CHIKV envelope and capsid proteins (Kramer et al., 2013).

Arévalo et al. (2019) reported 100% protection in adult mice against CHIKV infection when unadjuvanted CHIKV-VLPs were used. Similarly, Metz et al. (2013) compared three different vaccines in mice that were produced in insects by recombinant baculoviruses produced sE1, sE2 CHIKV, and CHIKV (VLPs). One-half of E1 and E2 immunized mice survived to show incomplete protection when compared with VLP-immunized mice.

Akahata and Nabel (2012) uncovered that variable CHIKV structural proteins expression results in VLPs, which mimic replication-competent alphaviruses. This vaccine caused the induction of neutralizing antibodies in large amounts against multiple strains of this virus and conferring complete protection. VLPs were easily produced from CHIKV 37997-strains compared to CHIKV-OPY-1 strain- VLPs despite high amino-acid-sequence similarity. Knowledge of mechanisms involved in CHIKV VLP production will help in other vaccine development also enhancing their range of application for other pathogens. Furthermore, VLP-production was affected by amino acid 234 of E2 in an acid-sensitive region. Mutations in the acid-sensitive region and pH changes also enhance the yield of VLPs.

### Influenza Virus

Influenza virus (Iv) infections are the leading cause of chronic human respiratory symptoms, leading to severe public health outcomes about endemic and seasonal infections and even result in unpredictable pandemic outbreaks (Lai et al., 2019). Iv is an enveloped, segmented, negative-sense RNA virus, which belongs to the family Orthomyxoviridae. To date, four types of Ivs have been identified, classified according to the presence of their core proteins: A, B, C, and D. Three Iv types, A, B, and C are pathogenic to human cells and cause severe infections, with Iv type A and B being the most prevalent circulating types. The main surface glycoproteins of influenza viruses are neuraminidase (NA) and hemagglutinin (HA) (To and Torres, 2019).

HA, the key antigen involved in infection, attaches to the residues of sialic acid on the cell membrane surface, facilitating the Iv to enter the host cell. NA appears to be less frequent on the viral surface as compared to HA, with a generally observed NA:HA ratio of 1:4. Its enzymatic function is critical in the cleavage of sialic acid, thereby facilitating viral release from the infected host cell surface. NA activation also enables the successful penetration by influenza to mucus through a mechanism involving cleavage of sialic (Buffin et al., 2019). Kim et al. (2019) investigated the cross-protective efficiency and immunogenicity of VLP containing NA (N1 VLP) derived from the 2009 H1N1 influenza viral pandemic and compared it to inactivated split influenza vaccine. Mouse immunized with the N1 VLPs was able to induce virus-specific Ab responses as well as cross-reactive NA inhibition activity, while strain-specific hemagglutination inhibition test was induced by inactivated split vaccination. Mice vaccinated with N1 VLPs led to the development of cross-protective immunity to antigenically various Ivs, as measured by changes in bodyweight, pulmonary viral titers, infiltration of innate leukocytes, cytokines and Ab secretory cells, and germinal center B-cells. Furthermore, in naïve mice, the immune sera of N1 VLPs conferred cross-protection. Immunity induced by N1 VLPs was neither impaired nor diminished in mice lacking the Fc receptor γ-chain. These findings indicate that NA representing VLPs, along with the current vaccination of influenza, may be further improved and exploited as an important candidate for cross-protective vaccines.

Recently, Kirsteina et al. (2020) studied and examined the efficacy and immunogenicity of an array of widely protective prototypes of Iv vaccine focused on both influenza triple matrix protein 2 ion channel (3M2e) and tri-stalk antigens incorporated into phage AP205 VLPs. VLPs that contained the 3M2e antigen alone stimulated protection in mice toward both standard homologous as well as heterologous virus challenge. The combination of both conserved antigens of the influenza virus into an individual VLP resulted in complete protection against a high dose of homologous influenza H1N1 infection in mice.

### Human Papillomaviruses

Human papillomaviruses (HPVs) are members of the Papillomaviridae family of tiny, circular, double-stranded DNA viruses that cause cervical cancer, the world’s second most fatal disease in women after breast cancer (Uddin et al., 2019; Burley et al., 2020). Currently, two VLP-based vaccines are commercially available for inhibiting warts and treating cervical cancers caused by HPV. These include Cervarix by GlaxoSmithKline (GSK) Pharmaceuticals and Gardasil by Merck Pharmaceuticals. Both vaccines consist of the immunogenic major CP (L1) VLPs of HPV 16 and 18, with Gardasil also having 6 and 11 VLPs (Uddin et al., 2019).

The drawback of using L1 protein as an antigen for the VLP vaccine is that it is not conserved among various HPV serotypes. Conversely, the minor CP (L2) is highly conserved across all HPV serotypes and has long been considered a major potential target antigen for developing an HPV vaccination with broad protection. In contrast to CP (L1), the CP (L2) cannot be used for VLP production and is, therefore, less immunogenic (Yadav et al., 2020). The reasons for the low immunogenicity of L2 proteins are due to their slight representation relative to L1, as well as the fact that L2 is mainly buried beneath the surface of the capsid, where it contacts the surface of the cell *in vitro* or the basement membrane *in vivo* and induces a conformational shift (Huber et al., 2017).

Several methods have been applied to improve and enhance the L2 peptides low immunogenicities, such as concatemeric fusion peptides consisting of L2 epitopes of various types of HPV, combined with immune activating TLR agonists or using repeated surface arrays of epitopes of L2 on particulate immunostimulatory platforms, such as VLP of TMV, bacteriophages MS2 or PP7, Adeno-associated virus, Lactobacillus casei, or bacterial thioredoxin (Huber et al., 2017).

Another promising strategy to improve L2 low immunogenicity is the presentation of L2 epitopes on the surface via VLPs assembled from HPV L1-L2 chimeric proteins. It was achieved through the genetic insertion of highly conserved B cell epitope RG1 of HPV16 L2 into the surface loop (DE) of the protein HPV16 L1 leading to its multivalent (360x) immunogenic display on the surface of VLP, whereas the conformational neutralization antigenic determinants of the HPV16 L1 scaffold remained mostly preserved. Immunizations with this chimeric RG1-VLP caused high type-specific HPV16 titers and extensive cross-neutralization of heterologous mucosal and distantly associated cutaneous HPVs (Schellenbacher et al., 2009, 2013).

Pouyanfard et al. (2018) described the thermostable thioredoxin vaccine development based on a single-peptide capable of carrying L2 polytopes from up to 11 various types of HPV. The antigens of the L2 polytope exhibit exceptional capabilities regarding the robustness and protection of the elicited immunological responses. To further boost and enhance immunogenicity, the polytope antigen L2 of thioredoxin was fused with a heptamerization domain. Protective responses to all 14 oncogenic types of HPV, as well as the lower risk HPV types (6 and 11), and a range of cutaneous HPVs were achieved in the final design of the vaccine.

## Virus-Like Particles Against Severe Acute Respiratory Syndrome-Coronavirus-2

With worldwide causalities crossing 2 million people and more than 20 million individuals affected in more than 200 countries this past year, the severe acute respiratory syndrome-coronavirus-2 (SARS-CoV-2) has emerged as a nuisance across the globe. The COVID-19 global pandemic resulting from SARS-CoV-2 has impacted billions of individuals and emerged as the first major global catastrophe after the 2009 H1N1-pandemic (Alvi et al., 2020). Currently, some commercially available antiviral drugs including Remdesivir^®^ (Gilead Sciences), Paxlovid^®^ (Pfizer), Molnupiravir^®^ (Merck and Co.) have been approved by the FDA and WHO for treatment of patients infected with SARS-CoV-2. Due to the limitations including development, cost, and distribution of targeted drug therapies against the virus, researchers are aiming their focus and efforts on developing vaccines for the long-term fight against COVID-19.

**FIGURE 5:**
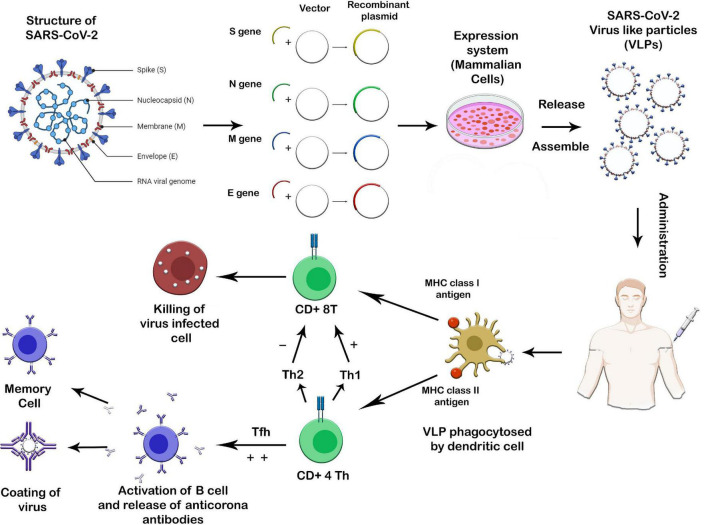
Proposed production system and mechanism of action of SARS-CoV2 virus-like particle vaccine. Plasmids encoding the structural proteins (S, N, M, and E) of the SARS-CoV2 can be transfected into an appropriate mammalian cell line. The assembled VLPs are then collected, purified, and administered to humans. The administration of VLPs stimulates both innate and adaptive immunological responses. If the original SARS-CoV2 enters the human body in the future, memory B cells activate and release antibodies against it. Similarly, the activated CD8+ T cells recognize and kill virus-infected cells.

VLPs against SARS-CoV-2 can help combat the widespread pandemic. VLPs can act as therapeutic agents and viable vaccines against this viral disease, with the addition of serving as a feasible diagnostic tool (Fuenmayor et al., 2017). SARS-CoV-2 has a slow mechanism of action as after entering host it takes between 5 and 15 days for a person to display symptoms because the virus enters host cells via endosomal pathway and is not efficient in evading the immune system whereas, vaccines against other coronaviruses (CoVs) strains that cause SARS and Middle East Respiratory Syndrome (MERS) were shown to be effective and active in animal models against SARS-CoV-2 (Cohen, 2020). Like other CoVs, SARS-CoV-2 consists of four proteins that are structurally conserved among different viral serotypes: Spike (S), Nucleoprotein (N), Envelope (E), and Membrane (M) proteins. The precise contribution of the above proteins and their relevant interaction patterns are crucial for the production and assembly of VLPs. M, N, and E are vital for the production VLPs for SARS-CoV-2 (Boson et al., 2020; Figure 5). The S-protein is one such protein that is common among different coronavirus strains and may serve as a potential vaccine development target. VLPs for MERS-CoV have been produced by displaying the S-protein in the insect host cells ES (Kato et al., 2019; Samrat et al., 2020).

Before developing SARS-CoV-2 VLP-based vaccines, previous work on VLPs against the preceding and closely related strains must first be considered. VLPs have previously been demonstrated to be effective in eliciting specific humoral and cellular immune defenses against SARS-CoV in mice by production in rBV ES (Lu et al., 2010). Ho et al. (2004) reported the synthesis of VLPs for SARS-CoV. These VLPS were assembled in the insect ES by the co-infection with rBV. VLPs prepared via the use of protein corona formation have shown effective vaccination and have led to the development of significant immune responses in the avian model of CoV infection (Chen et al., 2016). Similarly, plasmids containing 4 structural viral proteins (S, N, E, and M) of SARS-CoV were transferred into Vero E6 cells to produce VLPs. These VLPs may serve as an effective tool for studying the pathogenesis of SARS-CoV with the host cell (Hsieh et al., 2005). Recently, a COVID-19 mRNA vaccine expressing SARS-CoV-2 VLPs has shown the induction of a robust antiviral immunological response in the mouse model (Lu et al., 2020).

Currently, the NVX-CoV2372 is the only commercially available VLPs based vaccine against SARS-CoV-2. This vaccine is manufactured by the display of SARS-CoV-2 S-protein in rBV, which are then used for mass production by infecting moth cell expression system. This vaccine has shown various levels of effectiveness in different trials across separate countries (Heath et al., 2021; Shinde et al., 2021). Currently, five teams are also working separately on VLP based vaccines against COVID-19 (Bogani et al., 2020; Callaway, 2020), and clinical trials are underway with efforts being made in developing viable VLPs based vaccines. Biometric VLPs have been developed which can aid in accurate diagnosis of SARS-CoV-2 by acting as positive controls during RT-PCR procedure (Chan et al., 2020). Ghorbani et al. (2020) evaluated the immunogenic properties of various SARS-CoV-2 spike derived epitopes which had already been reported to induce a specific immunogenic response, by using immunoinformatic analysis. Finally, a set of screened epitopes were suggested for which a VLPs-based vaccines against SARS-CoV-2 could be synthesized in different plant species by using molecular farming approaches.

Different mammalian ESs were utilized for the synthesis of SARS-CoV-2 VLPs. Results indicate that SARS-CoV-2 VLPs produced from Vero E6 cell line are more durable and integrated than those derived from HEK-293T cells (Xu et al., 2020). Recently, a VLP vaccine based on plant ES against SARS-CoV-2 has been reported. Findings of clinical phase I trials of VLP vaccine (CoVLP) against SARS-CoV-2 have indicated significant development of IL-4 and IFN-γ immune responses in individuals. The CoVLP vaccine is manufactured by transient expression of S protein of SARS-CoV-2 in the plant ES (N. benthamiana). The trimeric S GPs are exhibited on the surface of self-assembling VLPs that imitate the size and shape of the SARS-CoV-2 (Ward et al., 2020, 2021). Similarly, a combination minispike VLP vaccine for SARS-CoV-2 has also demonstrated high level of immunization in mice after a single dosage. The vaccine elicited the development of neutralizing Abs and protected the K18-hACE2 mice from COVID-19 similar to the patients suffering from COVID-19 (Hennrich et al., 2021).

A capsid VLP centered SARS-CoV-2 vaccine (ABNCoV2) has shown efficient neutralization of SARS-CoV-2 in mice models. The ABNCoV2 vaccine is made by displaying the receptor-binding domain of the SARS-CoV-2 S-protein in insect (Drosophila) cells (Fougeoux et al., 2020). Researchers are also aiming toward developing a DNA-based vaccine that can be delivered through the nasal cavity to the targeted tissue, which will cause the production of SARS-CoV-2 VLPs resulting in a strong immune response in individuals (Samrat et al., 2020).

## Protein Nanoparticles for Vaccine Development

Protein nanoparticles (NPs) can help in the development of vaccines for immuno-evasive pathogens such as HIV, influenza, malaria, and can aid in the fight against emerging virulent strains by either acting alone or acting as carriers for targeted drug delivery (Hong et al., 2020; Roth et al., 2021). The engineering of protein molecules for antigen representation with the aid of NPs to be used in vaccine development for generating immune response is an increasingly popular and rapid field in therapeutic and drug development (Jain et al., 2018). Protein-based VLPs act as natural NPs that can be extensively used in vaccine development due to ease in design, self-assembly, and high stability (Nguyen and Tolia, 2021). Three different methods are used to attach antigens for presentation on NPs, (1) tag coupling (van Oosten et al., 2021), (2) chemical conjugation (Lu et al., 2021), and (3) genetic fusion (Antanasijevic et al., 2020). These techniques enable the platform to be decorated with a variety of antigens, leading to increased size and presentation (Nguyen and Tolia, 2021). Most antigens often do not self-assemble into NPs, like those utilized in influenza subunit vaccinations. Self-assembly can be achieved in such circumstances by attaching these antigens to an oligomeric protein platform (Nguyen and Tolia, 2021). There are many naturally occurring oligomeric proteins such as lumazine synthase (LS) (Ladenstein and Morgunova, 2020), ferritin (Rodrigues et al., 2021), dihydrolipoyl acetyltransferase (E2p) (He et al., 2016), non-structural protein 10 (nsp10) (Carter et al., 2020), encapsulin (Lagoutte et al., 2018), and heat shock proteins (HSPs) (Richert et al., 2012) that have been developed for platform design for designing targeted therapeutics. The display of viral glycoproteins using NPs is effective in developing antigen-specific antibodies (Ueda et al., 2020). Antigen-displaying protein NPs are highly effective for generating an immune response to specific antigens and can be used separately or in combination with vaccines (López-Sagaseta et al., 2016).

Among the wide array of platforms available for the display of antigens, ferritin has emerged as a major protein complex that can be combined with NPs for vaccine development (Diaz-Arévalo and Zeng, 2020). Due to its high pH and thermal stability, it allows for easy annealability with surface molecules (Rodrigues et al., 2021). In recent years, ferritin has been studied in preclinical studies as a viable vaccine platform for numerous viral infectious diseases, including HIV, H1N1, HCV, HBV, HFMD, Epstein–Barr, rotavirus, and respiratory diseases caused by coronaviruses (Butkovich et al., 2021). Kelly et al. (2020) compared the immunogenicity between ferritin NPs displaying influenza HA and soluble protein (HA) *in vivo*. HA-ferritin NPs showed higher immunogenicity and greater protection against a viral challenge as compared to soluble protein (HA).

Recently, a structure-based design of self-assembling protein NP immunogen that produces protective and potent antibody responses against SARS-CoV-2 has demonstrated the development of innate immunity in *in vivo* studies in mice (Walls et al., 2020). Similarly, a ferritin NP-based SARS-CoV-2 vaccine induced an effective antibody response in mice that reportedly lasted for at least 7 months post-immunization contributing toward sufficient development of immunity (Wang et al., 2021).

Though protein-NPs can serve as viable candidates for vaccine development, *in vivo* applications of NPs are often restricted by several challenges due to their organic nature (Poon and Patel, 2020), including cellular toxicity, inflammatory responses, and insufficient delivery to the target site (Kianfar, 2021). The term “protein corona” formation has been coined to summarize this unfavorable interaction between protein and NPs in *in vivo* conditions. The surface protein attached to NPs affects their biological behavior and changes their functionality, which occasionally results in gain or loss-of-function (Karch et al., 2018). The protein corona formation is a complex process that involves complex dynamics and kinetics between two interacting entities (Kapadia et al., 2019). Some cases have been reported where structural changes have occurred after interaction with NP surface evidently altering the NPs native function. Thus, to overcome such challenges it is critical to understand protein conformational changes and the unfolding process to accelerate the biomedical applications of NPs (Park, 2020).

## Conclusion

Over the past two decades, resistance in some pathogens against commercially available antimicrobial drugs has increased drastically, mainly due to unregulated over-the-counter sales, misuse and overuse of drugs, and genetic adaptations. The process of drug development is a rigorous task and requires ample time, funding, and repeated trials to commercialize a viable product that could aid in the fight against numerous emerging pathogens. Due to the time-consuming limitation, especially access of patients to appropriate drugs is hindered, whereas the resistance in pathogens is consistently increasing. Therefore, preventive measures are of significant importance to address the emergence of infectious diseases. In the efforts to combat such diseases, VLPs have come to light in the healthcare industry, with multiple applications ranging from vaccine development, drug delivery systems, and molecular diagnostics. VLP based approaches provide an alternative to the available conventional methods for vaccine developments. Currently, some VLP-based vaccines are commercially available against perilous pathogens like ZIKV, HCV, HBV, and HPV. Meanwhile, efforts are underway in the production and designing of efficient VLP vaccines against various emerging virulent pathogens, including SARS-CoV-2. However, there are still some major hurdles that need to be addressed before VLP-based therapeutics can compete with conventional drug therapies in terms of cost and effectiveness. Nonetheless, VLP-based medicinal strategies may be used extensively in the future along with pharmaceutics to aid in the fight against numerous infectious diseases.

## Author Contributions

HT and SB conceptualized and drafted the manuscript. HT, SB, and SA wrote the manuscript. HT was responsible for designing and illustrating the figures in the manuscript. MA reviewed and edited the manuscript. BA supervised and proofread the manuscript. All authors have read and agreed to the submitted version of the manuscript.

## Conflict of Interest

The authors declare that the research was conducted in the absence of any commercial or financial relationships that could be construed as a potential conflict of interest.

## Publisher’s Note

All claims expressed in this article are solely those of the authors and do not necessarily represent those of their affiliated organizations, or those of the publisher, the editors and the reviewers. Any product that may be evaluated in this article, or claim that may be made by its manufacturer, is not guaranteed or endorsed by the publisher.
